# More details, less variability? A crossover design study on the impact of information granularity on ChatGPT’s training program stability

**DOI:** 10.5114/biolsport.2026.154148

**Published:** 2025-09-29

**Authors:** Zhangyu Yang, Xing Zhang, Hansen Li, Jianfei Ye

**Affiliations:** 1 Department of Didactics of Body Expression, Faculty of Educational Sciences, University of Granada, Granada, Spain; 2 College of Physical Education, Fuyang Normal University, Anhui, China; 3 Department of Physical Education and Sport, Faculty of Sport Sciences, University of Granada, Granada, Spain; 4 School of Physical Education, Sichuan Agricultural University, Ya’an, China; 5 Institute of Physical Education, Huangshan University, Huangshan, China

**Keywords:** ChatGPT, Training program, Information granularity, Output variability, Artificial intelligence

## Abstract

This study aimed to evaluate how varying levels of information granularity affect the output variability and multidimensional quality — including Personality, Effectiveness, Safety, and Feasibility — of ChatGPT-generated training programs. A crossover design was used to compare simple and detailed input prompts, with each prompt input into GPT-4 (accessed via ChatGPT) four times to generate eight training programs. The training programs were anonymized by the research team and subsequently evaluated in a blinded manner by 11 experts (mean age = 35.4 years, average of 18.1 years of practical experience in the field of sport and exercise science). Output variability was assessed using the coefficient of variation (CV%), and quality ratings were based on a custom 15-item scale covering Personality, Effectiveness, Safety, and Feasibility. Differences in expert ratings across training programs were examined using repeated-measures ANOVA, with Friedman tests applied as sensitivity analyses to test the robustness of the results. Training programs generated from detailed input prompts consistently received higher expert ratings across all dimensions. CV% was generally lower under the detailed input prompts, indicating more stable outputs. Significant main effects of information granularity were found in Personality, Safety, Feasibility, and overall scores (all *p* < 0.05), though not in Effectiveness. Notably, repeated inputs of the same information granularity still yielded structurally and qualitatively different outputs, highlighting residual variability even under controlled conditions. Information granularity plays a crucial role in shaping the quality and stability of AI-generated training programs. Providing detailed, structured input enhances personalization, reduces output fluctuation, and improves alignment with exercise science principles.

## INTRODUCTION

It is widely acknowledged that physical activity is a safe and effective way to promote both mental and physical health [1]. These benefits include enhanced cognitive function [2], improved resilience [3], higher self-esteem [4], and reductions in stress, depression, and anxiety symptoms [5, 6], as well as a decreased risk of cardiovascular disease and type 2 diabetes [7]. However, to maximize the physical and psychological benefits, effective training program is essential. As proposed in prior work [8, 9], an effective training program meets individual needs and goals and elicits training-specific responses and adaptations by appropriately manipulating variables such as the FITT principle (Frequency, Intensity, Time, and Type). Ineffective training programs may lead to adverse outcomes [10, 11]. For instance, training programs lacking appropriate progression and individualization resulted in increased injury rates among athletes [12]. Similarly, excessive or abrupt increases in exercise intensity and volume led to higher risks of musculoskeletal injuries and exercise dropout in children [13] and community-dwelling women [14]. Moreover, insufficient exercise intensity may fail to induce necessary physiological adaptations, thereby limiting the effectiveness of the intervention [15].

To design an appropriate training program, individuals require specialized knowledge in exercise science or sports rehabilitation [16, 17], including an understanding of feasibility, overload, and individualization—components widely regarded as critical for program quality and exercise adherence [18]. However, in addition to limited expertise and financial barriers [17], individuals often face other obstacles that hinder regular engagement in physical activity—such as time constraints, lack of motivation, or limited access to suitable facilities [19]. These multifactorial barriers underscore the need for scalable, low-cost tools that can provide accessible and individualized exercise guidance. In this context, ChatGPT may offer potential value as an on-demand, user-friendly platform that may help reduce both the knowledge and psychological barriers to accessing effective training programs.

Generative artificial intelligence (AI), particularly large language models (LLMs), represents a transformative class of tools capable of producing coherent, human-like responses across a wide range of domains [20, 21]. LLMs, such as GPT-4, DeepSeek, and Google Gemini, are advanced neural network architectures trained on massive corpora of text using self-supervised learning techniques [22, 23]. These models underpin various chatbot applications, including ChatGPT, which serves as a widely adopted user-facing interface built on top of the GPT-4 model. Among these, ChatGPT is a prominent example, widely adopted for its ability to generate contextually relevant and conversational outputs in response to user input on specific topics or fields of inquiry [24]. One of its key advantages lies in its capacity to process and synthesize large volumes of textual data within a short time frame, thereby significantly reducing users’ time and cognitive effort [20]. Owing to its accessibility, open-source availability, and ease of use, GPT-4 has rapidly been adopted across diverse domains, including education [25], medicine and healthcare [26]. Notably, it has also been proposed as a potentially useful tool for reducing barriers associated with limited exercise knowledge and financial constraints [17]. Preliminary evidence has begun to validate the utility of ChatGPT in generating training programs, including its application in designing rehabilitation programs for older adults [16], creating running plans for recreational runners [27], offering exercise advice for individuals aiming to lose weight [17, 28], and even developing individualized exercise recommendations for patients with hypertension [9]. Moreover, recent work has systematically reviewed the emerging applications of generative AI in sport and exercise science, emphasizing both the promise of such tools and the methodological limitations that currently constrain their real-world implementation. This highlights the need for empirical investigations into how a tool like ChatGPT performs in specific training program contexts and under varying user input conditions [29].

As research on ChatGPT’s performance in training programs continues to evolve, concerns have also emerged—particularly regarding the variability of its outputs. In principle, ChatGPT operates using probabilistic prediction mechanisms that generate responses word by word based on user input [30, 31]. Since multiple tokens may have similar probabilities of selection, the model samples among high-probability candidates to enhance linguistic diversity and naturalness in its output [32]. As a result, when users request training programs using the same ChatGPT model, it is possible to receive different outputs from identical inputs. This raises concerns about the consistency and reliability of such outputs—especially given that both acute responses and long-term adaptations to exercise are highly sensitive to variables such as exercise type, intensity, volume, and rest intervals [33].

To address concerns regarding the reliability of AI-generated training plans, the extant literature includes only one empirical study that has specifically investigated their reproducibility. This prior work primarily focused on inter-model comparisons (i.e., GPT-4 vs. Google Gemini) and found that the quality of hypertrophy-related training programs improved with more detailed input information [34]. Notably, however, that study examined only two output attempts per model and did not disentangle the effects of input repetition from prompt granularity. As such, the extent to which different levels of informational granularity affect output variability remains underexplored. To bridge this gap, we did not aim to evaluate the accuracy or clinical appropriateness of the training programs, but rather to examine their variability and quality across different input conditions.

## MATERIALS AND METHODS

### Output Variability and Information Granularity

To explore the output variability of AI chatbot–generated training programs, each protocol prompt was input four times in this study. The two protocols were conducted on separate days, with each input attempt spaced 10 minutes apart. To minimize potential interference from the model’s contextual memory, each attempt was conducted in a freshly initiated dialogue window with an explicit instruction to disregard previous conversations.

Users differ in their sports science knowledge and exercise experience, which affects the information they provide when interacting with AI chatbots. To reflect this, we developed two protocols: simple input and detailed input information. These were designed to simulate users with and without relevant expertise or experience. Based on previous studies, our protocols are detailed as follows [17, 27].

– Protocol 1: *Please design a one-month training program for my 15-year-old son aimed at weight loss and general fitness.*
– Protocol 2: *My son is 15 years old, 175 cm tall, and weighs 75 kg. He is healthy, with no history of surgery or chronic illness. At school, he enjoys playing basketball and running. Please create a one-month training program focusing on weight reduction and physical fitness enhancement. The plan should follow the FITT principle, specifying frequency, intensity, time, and type of exercise. Make sure the exercise types are age-appropriate and suitable for his health status, and include 2–3 alternative exercise options to ensure variety. Present the plan in a table format, seamlessly integrating weekdays and rest days, along with relevant annotations where necessary.*

### Study Design

This study employed a crossover design to examine the impact of varying information granularity on the variability and quality of training programs generated by an AI chatbot. Previous research has conceptualized high-quality training as a multifaceted process involving not only exercise content but also preparation, execution, and recovery phases, such as adequate rest, personalized programming, intensity regulation, and post-exercise reflection [8]. Drawing on this framework, the current study assessed output quality across four key dimensions—Personality, Effectiveness, Safety, and Feasibility—which collectively reflect both the scientific validity and practical applicability of the generated training programs. To achieve this, the researchers recruited a real-life parent of a junior high school student with no formal background in exercise science or AI. This individual was instructed to input prompts based on their natural language and personal intentions, reflecting how a typical layperson might interact with ChatGPT to generate a training program. This recruitment strategy also helps fill a gap in prior research, which often focuses on hypothetical individuals presenting with only one health condition [25, 27, 35], by incorporating real-world user characteristics to better reflect the complexities of practical training program scenarios. To minimize individual-level confounding and enhance within-subject comparability, the same user generated exercise prompts under both Protocol 1 (simple prompt) and Protocol 2 (detailed prompt), with each protocol repeated four times. To avoid carryover effects across conditions, each prompt interaction was conducted in a newly opened ChatGPT session with no access to prior history. The user was explicitly instructed to clear any existing conversation threads and to initiate each session from a fresh window to minimize memory persistence or contextual overlap. The order of conditions was fixed, and all prompt-response interactions were conducted independently to maintain condition-specific integrity. All generated outputs were compiled and formatted in Excel, anonymized through coded labeling, and subsequently evaluated by an expert panel in a blinded rating process. The resulting scores were used for the final statistical analysis. This study was conducted in accordance with the Declaration of Helsinki and was approved by the Ethics Review Committee of [blinded for peer review].

### Participant

A real-life family, consisting of a junior high school student and his mother, was recruited through online advertising to participate in this study. Exclusion criteria included any history of serious musculoskeletal disorders, cardiovascular disease, or other medical limitations to exercise. The family had no prior involvement in research related to exercise science or AI applications. At the outset of the experiment, relevant background information was collected and used to inform the personalized prompt. The mother served as the sole prompt provider to simulate a real-world scenario in which a caregiver seeks exercise advice for their child using generative AI. Prior studies have identified parents as key mediators of youth physical activity behaviors and frequent users of online health information sources, especially when professional guidance is unavailable or inaccessible [36]. The mother’s lack of formal expertise in exercise science or artificial intelligence was intended to reflect typical layperson interaction patterns with generative AI. These data included the participant’s gender, age, height, weight, current health status, medical history, prior training experience, personal interests, and primary motivation for engaging in physical activity (Table 1).

**TABLE 1:** Basic Information of Participants

Category	Basic Information
	**Participants’ Mother**
Gender	Female
Age	35 years old

	**Child's information**
Age	15 years old
Height	175 cm
Weight	75 kg
Health status	Healthy
Injury history	None
Exercise experience	None
Hobbies	Basketball/Running
Exercise goal	Weight loss and improved fitness

These details were collected for two main purposes: first, to define the characteristics of the participant and clarify the target population to which the findings may be applicable; and second, to provide the personalized information necessary for detailed prompts used to generate individualized training programs. Informed consent was obtained from participants before initiating the procedures.

### Procedures

This study was conducted using the ChatGPT-4.0 model between March 23 and 29, 2025. In the first phase, a researcher guided the participant’s mother in completing input protocols, which were administered in a fixed sequence (Protocol 1 followed by Protocol 2). In the second phase, the first researcher collected all AI-generated training programs and organized them into an Excel spreadsheet. A second researcher independently reviewed the training programs to verify their completeness and accuracy. Any discrepancies identified during the review process were resolved through discussion between the two researchers, with a third researcher consulted when necessary. In the third phase, an expert panel conducted a blind evaluation of the eight generated training programs. To eliminate potential evaluation bias, all training programs were anonymized and recoded with non-indicative identifiers (labeled 1 through 8) prior to presentation to the experts. Finally, upon completion of expert ratings, the first researcher handled data entry and preliminary validation, while the second researcher performed a secondary review to ensure data accuracy and integrity.

### Expert Panel Selection

To ensure that the expert panel had a solid theoretical foundation, the following inclusion criteria were applied: (1) Held a doctoral degree in sport science or a related discipline, had obtained a professorial title, or were currently pursuing a PhD in a sport-related field; (2) Held a master’s degree and had at least five years of teaching or research experience in sport science; (3) Actively engaged in sport science–related occupations (e.g., physical education instructors or strength and conditioning coaches) with no less than five years of front-line teaching or professional training experience. Individuals who fulfilled any of the above criteria were considered eligible for participation in the expert review panel.

### Instruments and Measurements

To comprehensively and systematically assess the quality of the AI chatbot-generated training programs, a customized evaluation scale was developed based on prior literature and the specific needs of the present study [17, 34]. The scale comprised four core dimensions: Personality, Effectiveness, Safety, and Feasibility, encompassing a total of 15 items. Each item was rated using a 5-point Likert scale (1 = strongly disagree to 5 = strongly agree). Scores for each dimension were calculated as the average of their respective items, and the total score was derived by summing the scores across all four dimensions. Details of the rating scale are provided in Table 2.

**TABLE 2:** Scale for evaluating the quality of training programs

Dimension	No.	Item Description	Rating (1 = Strongly Disagree, 5 = Strongly Agree)
Personality	1	Does the exercise prescription align with the individual’s training goals (e.g., weight loss, muscle gain, improved cardiorespiratory endurance)?				

	2	Is the exercise prescription tailored to the individual’s characteristics (e.g., age, gender, prior training experience)?				

	3	Does the exercise prescription take into account the individual’s health status (e.g., current conditions and medical history)?				

	4	Does the exercise prescription incorporate personal preferences and consider potential barriers to long-term adherence?				

	5	Is the exercise prescription flexible enough to be adjusted and optimized based on individual needs?				

Effectiveness	6	Are the training variables appropriately designed (e.g., frequency, intensity, training volume, and recovery time)?				

	7	Does the exercise prescription provide sufficient training stimulus to achieve the stated goals?				

	8	Does the exercise prescription follow established training principles (e.g., specificity, recovery, progressive overload)?				

	9	Does the exercise prescription include methods or criteria to track and monitor training status (e.g., heart rate, RPE scale)?				

Safety	10	Does the exercise prescription comply with recognized safety guidelines and best practices (e.g., ACSM, WHO)?				

	11	Does the exercise prescription include necessary technique instructions, precautions, and monitoring methods to reduce injury risk?				

	12	Does the exercise prescription adequately consider individual health risks (e.g., chronic conditions, previous injuries), and provide appropriate modifications?				

### Statistical Analysis

To evaluate score dispersion between two protocols, the withinsubjects coefficient of variation (CV%) was calculated as the ratio of the standard deviation to the mean (CV = SD/Mean × 100), reflecting the variability of training programs generated by an AI chatbot.

Descriptive statistics are reported as means and standard deviations (SD). The normality of the data was assessed using the Shapiro–Wilk test. To explore whether the experts’ professional backgrounds influenced their evaluations, expert ratings between the PE and non-PE groups were compared using independent Wilcoxon rank-sum tests. To examine whether the ratings differed significantly across training programs and protocols, a two-way repeated-measures ANOVA was conducted (protocols × training programs). Mauchly’s test of sphericity was applied, and when violated, Greenhouse-Geisser correction was reported. Post hoc analysis was conducted using paired-sample t-tests, with Bonferroni correction applied for multiple comparisons. Given that the Feasibility dimension under the detailed protocol violated the assumption of normality, non-parametric Friedman tests were conducted as a sensitivity analysis to verify the robustness of the findings.

All statistical analyses were conducted using R software (version 4.3.1; R Core Team, Vienna, Austria) in RStudio (version 2023.06.1+524; RStudio, PBC, Boston, MA, USA). The main analyses were conducted using the rstatix and ezANOVA package. A *p*-value of < 0.05 was considered statistically significant.

## RESULTS

### Characteristics of Expert Panel

The expert evaluation panel comprised 11 professionals (mean age: 35.4 ± 7.5 years; mean practical experience: 18.1 ± 7.3 years) with formal academic backgrounds in sport and exercise science. All members had completed sports-related university education and possessed substantial practical experience in physical education, athletic training, or related disciplines. The panel included two senior experts holding either a doctoral degree or professorial title, two doctoral candidates specializing in exercise science, five frontline physical education teachers with master’s degrees, and two strength and conditioning specialists with extensive field-based and academic training in sport science. The detailed information is provided in Supplement 1. To explore whether the professional background of experts influenced their evaluations, the expert panel was divided into two groups based on their area of expertise: Physical Education (PE) and non-Physical Education (non-PE). As shown in Supplement 2, independent Wilcoxon rank-sum tests revealed that none of the comparisons between the two groups reached statistical significance after Bonferroni correction (all adjusted *p* > 0.05).

### Output Variability

The present study examined the variability of AI chatbot-generated training programs under different information granularity (Table 3). Results indicated that CV% values were generally higher for Simple prompts than for Detailed prompts in dimensions such as Personality (8.40% vs. 7.79%), Feasibility (7.24% vs. 5.63%), and Safety (9.83% vs. 7.88%). However, the opposite pattern was observed in dimensions like Total (5.97% vs. 6.41%) and Effectiveness (8.56% vs. 11.20%), where Simple prompts yielded lower variability. Post hoc analysis revealed no statistically significant difference in overall CV between the two protocols (*p = 0.814*).

**TABLE 3:** Summary of mean ratings and CV% of AI chatbot-generated training programs

Protocol	Dimension	Training Programs (Mean ± SD score)					Anova *p*	CV (%)

		1	2	3	4	Overall		
Simple	Total	14.69 ± 2.92	14.71 ± 2.95	15.21 ± 2.59	14.78 ± 3.18	14.8 ± 2.82	0.49	5.97
Simple	Personality	3.67 ± 0.83	3.65 ± 0.75	3.89 ± 0.55	3.65 ± 0.9	3.72 ± 0.75	0.309	8.4
Simple	Effectiveness	3.68 ± 0.84	3.55 ± 0.8	3.77 ± 0.75	3.7 ± 0.89	3.68 ± 0.80	0.395	8.56
Simple	Safety	3.24 ± 1	3.3 ± 1.05	3.45 ± 0.86	3.33 ± 1	3.33 ± 0.95	0.517	9.83
Simple	Feasibility	4.09 ± 0.79	4.21 ± 0.64	4.09 ± 0.72	4.09 ± 0.76	4.12 ± 0.71	0.842	7.24
Detailed	Total	15.92 ± 2.75	17.23 ± 1.84	17.09 ± 1.78	17.05 ± 2	16.8 ± 2.12	0.048*	6.41
Detailed	Personality	3.96 ± 0.66	4.4 ± 0.55	3.25 ± 0.57	4.24 ± 0.54	4.21 ± 0.58	0.043*	7.79
Detailed	Effectiveness	3.77 ± 0.95	4.23 ± 0.54	4.23 ± 0.51	4.05 ± 0.62	4.07 ± 0.81	0.136	11.2
Detailed	Safety	3.79 ± 0.64	4.09 ± 0.6	3.94 ± 0.59	4 ± 0.67	3.95 ± 0.61	0.234	7.88
Detailed	Feasibility	4.39 ± 0.84	4.52 ± 0.58	4.67 ± 0.42	4.77 ± 0.35	4.59 ± 0.58	0.052	5.63

Note: The *p*-values reflect statistically significant differences among the four training programs within each dimension (*, *p* < .05). CV (%) refers to the coefficient of variation.

**TABLE 4:** Results of two-way repeated-measures ANOVA

Dimension	ANOVA		

	Effect	F	*p*
Personality	Protocols	7.59	0.02^*^
	Training Programs	1.61	0.208
	Protocols × Training Programs	3.56	0.026*

Effectiveness	Protocols	4.48	0.06
	Training Programs	1.17	0.337
	Protocols × Training Programs	2.54	0.075

Safety	Protocols	6.46	0.029*
	Training Programs	1.03	0.396
	Protocols × Training Programs	1.42	0.257

Feasibility	Protocols	5.98	0.035*
	Training Programs	0.95	0.429
	Protocols × Training Programs	1.77	0.174

Total	Protocols	8.62	0.015*
	Training Programs	1.71	0.187
	Protocols × Training Programs	2.73	0.062

Note:

* ,

p < 0.05

### Results of Statistical Analysis

#### Analysis of Training Programs Generated by Simple Protocol

The overall mean scores across the evaluated dimensions were as follows: 3.72 ± 0.75 for Personality, 3.68 ± 0.80 for Effectiveness, 3.33 ± 0.95 for Safety, 4.12 ± 0.71 for Feasibility, and 14.8 ± 2.82 for the total score (Table 3). No significant main effect was observed among the four training programs generated within the same dimension under the simple protocol (*p* > 0.05).

#### Analysis of Training Programs Generated by Detailed Protocol

The overall mean scores across the evaluated dimensions were as follows: 4.21 ± 0.58 for Personality, 4.07 ± 0.81 for Effectiveness, 3.95 ± 0.61 for Safety, 4.59 ± 0.58 for Feasibility, and 16.8 ± 2.12 for the total score (Table 3). Initial statistical analyses revealed that significant main effect was present in only two dimensions—Total (*p* = 0.048) and Personality (*p* = 0.043)—while no significant main effect was observed in the remaining dimensions (*p* > 0.05) among the four training programs generated. However, post-hoc comparisons indicated no significant differences in scores among the four training programs (*p* > 0.05).

#### Comparison Between Simple and Detailed Protocols

**FIG. 1:**
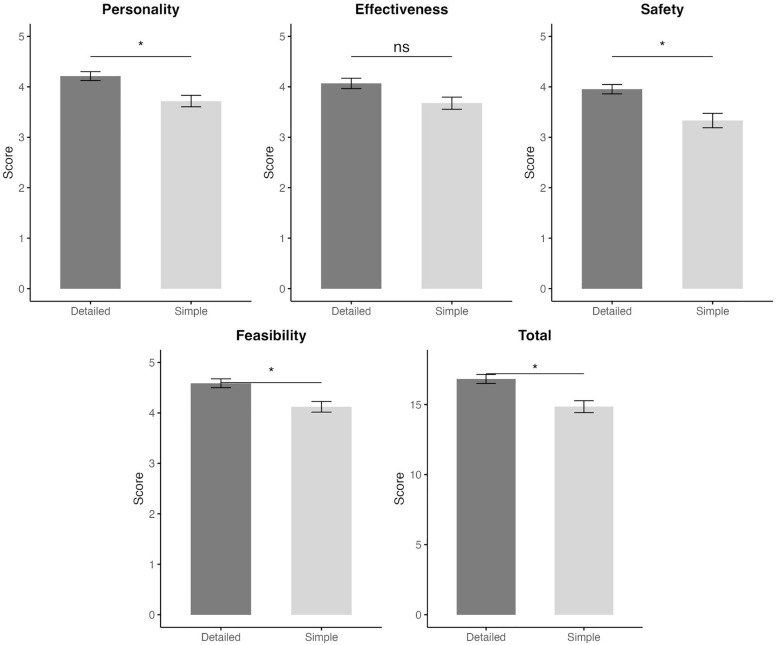
Comparison of mean expert ratings between Simple and Detailed protocols across assessed dimensions. Asterisks indicate significant main effects based on repeated-measures ANOVA (*, *p* < 0.05); ns = not significant.

These findings are visually summarized in Figure 1, which illustrates the mean expert ratings across dimensions under the Simple and Detailed protocol conditions. A two-way repeated measures ANOVA was conducted for each dimension to examine the main and interaction effects of protocols (Simple vs. Detailed) and training programs (1 to 4) on expert ratings. Significant main effects of protocols were observed in Personality (F_(1, 10)_ = 7.59, *p* = 0.020), Safety (F_(1, 10)_ = 6.46, *p* = 0.029), Feasibility (F_(1, 10)_ = 5.98, *p* = 0.035), and Total (F_(1, 10)_ = 8.62, *p* = 0.015). No significant main effects or interaction effects were observed in Effectiveness (all *p* > 0.05). A significant interaction effect between protocols and training programs was found in the Personality dimension (F_(3, 30)_ = 3.56, *p* = 0.026).

#### Sensitivity Analysis

In the initial repeated-measures ANOVA, no significant differences were observed among the four training programs in the Feasibility dimension under the Detailed protocol (*p* > 0.05). However, as this dimension violated the normality assumption, a non-parametric Friedman test was conducted as a sensitivity analysis to ensure the robustness of the findings. The Friedman test yielded consistent results, showing no statistically significant differences after Bonferroni correction (adjusted *p* > 0.05). The results obtained from both parametric and non-parametric analyses were consistent and the detailed information is provided in Supplement 3.

## DISCUSSION

### Overview of the Study and Main Findings

To the best of our knowledge, this study is the first to apply a repeated input design to examine how output variability, influenced by information granularity, affects the quality of AI-generated training programs. The findings revealed that, with the exception of the Effectiveness dimension, the coefficient of variation (CV%) for the remaining three dimensions under the simple protocol was consisuncertainty and instability in outputs generated from less specific input. Information granularity was found to significantly influence expert ratings on Personality, Safety, Feasibility, and Total score dimensions, while no significant effect was observed in Effectiveness. These results collectively underscore the advantage of detailed information in enhancing output consistency and content completeness in AI-generated training programs.

### Output Variability

The completeness and granularity of user-provided information can vary considerably, which may directly impact the quality and consistency of AI-generated outputs, thereby affecting their applicability in health-related training programs. The results of this study demonstrated that, across most evaluation dimensions, training programs generated using a detailed protocol exhibited lower CV%, indicating reduced fluctuation in expert ratings and greater output stability. This disparity may be attributed to the structural differences in input information granularity. Specifically, the simple protocol tends to lack essential individual background information and fails to guide the generation of tailored training programs based on established frameworks such as the FITT principle. As a result, the language model produces outputs with reduced consistency and comparability, leading to more dispersed expert evaluations [27, 34]. These findings underscore the importance of structured prompt inputs. For users without formal training in exercise science, supplying more specific and well-organized information may enhance the quality and relevance of AI-generated training programs, reducing the need for repeated interactions and compensating for the absence of professional guidance. This approach not only enhances the efficiency of obtaining reliable exercise guidance—by reducing the need for repeated interactions and prompt refinements—but also helps mitigate potential health risks associated with inconsistent or inaccurate AI-generated recommendations [37]. For instance, Rocha-Silva et al. demonstrated that both GPT-3.5 and GPT-4o initially provided inaccurate explanations of exercise-induced fatigue, such as attributing fatigue solely to lactate accumulation, which oversimplifies the underlying mechanisms. Only after multiple user prompts and corrections did the model generate more scientifically accurate responses [38]. This example illustrates how vague or unstructured prompts may lead to misinformation. By contrast, structured, detailed input from the outset can therefore reduce the likelihood of misinformation and improve the safety of AI-generated training programs [34].

Interestingly, in the Effectiveness and Total dimensions, the CV% was slightly higher under the detailed protocol compared to the simple one. This unexpected pattern may be attributed to the increased complexity of the content generated under the detailed protocol. Specifically, detailed prompts often yield outputs that include a broader array of training components such as exercise selection strategies (e.g., compound vs. isolation movements), periodization or progression schemes, intensity and volume training programs, and instructional cues. These diverse content elements may lead experts to evaluate the scientific rigor and applicability of the program from different perspectives, increasing variability in ratings. In particular, discrepancies may arise when experts emphasize different aspects such as training frequency, intensity distribution, or exercise modality. Even within a specific training goal—such as hypertrophy—multiple evidencebased approaches exist, including both low-load (30–50% 1RM with higher repetitions) and high-load (70–85% 1RM with moderate repetitions) training protocols [39]. Experts may differ in their appraisal of these methods based on their theoretical orientation or practical experience, contributing to variation in perceived effectiveness [34]. Furthermore, since Total represents a composite score across all dimensions, even small inconsistencies in subdimension ratings can be statistically amplified, resulting in a higher overall CV%.

It is also noteworthy that, even when the level of information granularity was held constant, AI-generated training programs still displayed considerable differences in structure, content detail, and domain-specific rigor. Although no statistically significant differences were detected among the four training programs generated under repeated inputs of identical information granularity, meaningful variation was observed in aspects such as exercise selection, intensity training program, and degree of personalization. For instance, under the simple protocol—e.g., “Please design a one-month weight-loss and fitness plan for my 15-year-old son”—ChatGPT often failed to produce a fully structured and actionable training program. Instead, the AI chatbot frequently responded with clarifying questions, requesting additional information on the individual’s height, weight, health status, schedule availability, and academic workload (Supplement 4). Although such behavior did not occur in every instance, it underscores the model’s high dependency on input completeness and contextual cues when tasked with generating structured outputs. This observation aligns with findings from prior studies, even those involving different AI platforms (e.g., Google Gemini), which report similar tendencies: when presented with vague or incomplete prompts, language models are more likely to request supplementary information rather than generate concrete recommendations [34]. Collectively, these results reinforce the notion that detailed information inputs are essential for optimizing the completeness, relevance, and practical utility of AI-generated training programs.

### Output Quality

This study found that training programs generated using detailed protocol consistently received higher expert ratings across all evaluation dimensions compared to those generated with simple protocol. This finding aligns with previous literature suggesting that the quality of training recommendations improves with the provision of more comprehensive input information [27, 34, 40]. In the present study, the detailed protocol incorporated a range of participant-specific characteristics, including height, weight, injury history, and exercise preferences. As a result, ChatGPT was able to produce more tailored and individualized content. For example, under the detailed protocol, many of the generated training programs explicitly integrated preferred physical activities such as basketball or running—elements that likely enhanced both user engagement and the long-term feasibility of implementation. Although the simple protocol lacked such individualized input, often resulting in more generic and abstract outputs that were less aligned with specific user needs, the generated programs still adhered to fundamental exercise principles. They typically included at least two resistance training sessions per week, incorporated aerobic components, and followed a structured format with warm-up, main exercise, and cool-down phases—aligning with WHO recommendations for moderate-intensity physical activity (e.g., ≥ 150 minutes/week) [41]. These training programs generated by the simple protocol may be useful for users without formal training in exercise science; however, their real-world effectiveness requires validation through future experimental research.

With the exception of the Effectiveness dimension, prompt granularity had a significant influence on the quality of AI-generated training programs, with detailed protocols consistently outperforming simple ones. While the Effectiveness dimension did not demonstrate a statistically significant difference, the observed marginal trend indicates that it may still be responsive to variations in input information. This finding further reinforces the critical role of information granularity in influencing output quality. Notably, the detailed protocol in this study explicitly instructed the AI chatbot to design training programs in accordance with the FITT principle—Frequency, Intensity, Time, and Type—which guided ChatGPT to generate content with greater scientific rigor and internal consistency [17, 18]. This was particularly evident in how training load and activity types were structured more systematically and purposefully. In the Safety dimension, training programs generated under detailed protocol tended to incorporate reasonable adjustments to exercise intensity and duration based on activity type, potentially reducing the risk of exercise-related injuries.

These findings highlight the importance of information granularity in influencing the quality, completeness, and individualization of AI-generated outputs. The observed advantages of detailed protocol may stem from the language model’s enhanced ability to interpret and operationalize structured, context-rich inputs [42]. When users provide clearly defined goals, health conditions, preferences, and constraints, the model is better positioned to generate tailored, coherent, and practically relevant training programs that align more closely with user expectations [43].

### Practical Considerations and Cautions

Although AI tools such as ChatGPT show promise in supporting the design of training programs, caution must be exercised to avoid over-reliance on their outputs. First, in practice, the training plans displayed in the ChatGPT interface sometimes differed from the versions available for download (see Supplement 5), highlighting a potential risk of output inconsistency. This underscores the need for users to carefully verify the accuracy of the generated content before application. Second, this study identified potential issues related to the memory architecture of large language models. Despite initiating each protocol in a newly opened window to minimize contextual interference, residual memory effects may still persist across sessions, potentially influencing subsequent outputs. This observation suggests that future studies involving multi-round AI interactions should incorporate explicit strategies for resetting model memory to ensure the independence and reliability of input–output processes.

Beyond technical issues such as output inconsistency and memory persistence, recent studies have raised broader concerns about the limitations of AI-generated health content, particularly in terms of safety, individualization, and evidence transparency. For example, GPT-based models often default to overly conservative training programs, lack nuanced progression schemes, and fail to sufficiently adapt to individual health needs or real-time physiological feedback— especially for users with chronic conditions, comorbidities, or mental health challenges [35, 40]. Specifically, in resistance training, GPT-generated programs—while generally aligned with broad scientific principles—have been shown to overlook individualized periodization strategies and emerging methods such as blood flow restriction or cluster sets [44].

To maximize the utility of AI-generated training programs while minimizing risks, users should consider the following prompt engineering strategies:

1. Specify key personal attributes: Including age, sex, height, weight, health status, prior training experience, and physical activity goals helps the model generate more individualized and safer programs;
2. se structured language: Clearly label dimensions such as frequency, intensity, time, and type (i.e., the FITT principle) to encourage the model to adhere to evidence-based frameworks;
3. Include safety-related constraints: For example, stating “no previous injuries, but please ensure the plan avoids high-impact movements” can reduce the risk of inappropriate recommendations;
4. Avoid vague or overly broad prompts: General queries such as “Give me a training plan” often yield generic and less actionable responses, as also observed in this study’s “simple” condition.

Taken together, while chatbot interfaces powered by LLMs such as GPT-4 (e.g., ChatGPT) may assist in generating draft-level or entry-level training programs, they cannot currently substitute for the expert judgment of qualified professionals [29]. As generative AI becomes more integrated into health practice, future applications must prioritize accountability, dynamic interactivity, and evidence-based refinement.

## LIMITATIONS

Several limitations of this study should be acknowledged and addressed in future research. First, this study relied on a single user and focused solely on training programs generated for a single adolescent population. This limited sample restricts the generalizability of the findings, as results may not apply to other age groups, user demographics, or AI platforms. Future studies should involve more diverse users and contexts to assess the broader applicability of these outcomes. Second, to simulate naturalistic usage, prompts were submitted on different days. However, it is possible that internal model adjustments or selfcalibration processes may have occurred, introducing potential risks of output drift. This temporal variability should be considered when interpreting the findings. Third, the study’s generalizability is limited by several methodological constraints. The evaluation relied on a relatively small panel of expert raters, whose subjective judgments may introduce bias. Additionally, the analysis was restricted to a single large language model (GPT-4), which may not reflect the variability in the quality and reasoning of outputs across other generative AI platforms. Future research should involve multiple user profiles, a wider range of prompt types, and comparative evaluations across different LLMs to strengthen external validity. Fourth, the study was conducted using GPT-4, which is no longer widely available. As a result, our findings may not be fully reproducible with newer versions (e.g., GPT-4o, GPT-5), and the main contribution should therefore be regarded as methodological rather than model-specific. Finally, the evaluation scale used to assess the quality of AI-generated training programs was developed specifically for this study and has not yet undergone formal validation in terms of reliability and construct validity. As such, there is a potential risk of measurement bias. Future studies are encouraged to develop and validate standardized instruments for evaluating AIgenerated training programs, in order to enhance the scientific rigor and reliability of outcome assessments.

## CONCLUSIONS

This study investigated the impact of information granularity on the variability and stability of training programs generated by ChatGPT. For adolescents, providing structured and detailed information inputs can significantly improve the efficiency and quality of training programs, even without professional guidance. Such practices may reduce the need for repeated interactions and mitigate potential health risks associated with inconsistent or generic AI-generated content.

## Supplementary Material

More details, less variability? A crossover design study on the impact of information granularity on ChatGPT’s training program stability

## Data Availability

The data collected in this study will not be publicly available. However, the corresponding author can be contacted for de-identified data on reasonable request.
