# Supplementary material for: More details, less variability? A crossover design study on the impact of information granularity on ChatGPT’s training program stability
Source: Biol Sport. 2025 Sep 29;43:379–92. doi: 10.5114/biolsport.2026.154148 (PMC12884907; doi:10.5114/biolsport.2026.154148)
Supplement: More details, less variability? A crossover design study on the impact of information granularity on ChatGPT’s training program stability [file JBS-43-56667-s1.pdf]

## SUPPLEMENTARY MATERIAL

### SUPPLEMENT 1

| Expert ID | Gender | Academic Degree | Age | Area of Expertise | Years of practical experience in the field of sport and exercise science |
|---|---|---|---|---|---|
| E1 | Male | Master degree in Sport Science | 27 | Exercise Training | 10 |
| E2 | Male | PhD Candidate in Physical Education | 35 | Physical Education | 18 |
| E3 | Male | Master degree in Physical Education | 33 | Physical Education | 15 |
| E4 | Male | Master degree in Physical Education | 35 | Physical Education | 18 |
| E5 | Male | Master degree in Sport Training | 35 | Exercise Training | 18 |
| E6 | Male | Strength & Conditioning Coach | 28 | Physical Education | 11 |
| E7 | Male | PhD Candidate in Physical Education | 35 | Strength & Conditioning | 18 |
| E8 | Male | Professor in Sport Training | 56 | Sport Training | 38 |
| E9 | Male | PhD in Physical Education | 34 | Exercise Training | 16 |
| E10 | Male | Master degree in Physical Education | 35 | Physical Education | 18 |
| E11 | Male | Strength & Conditioning Coach | 36 | Strength & Rehab Training | 19 |

**SUPPLEMENT 2.** Results of Wilcoxon Rank-Sum Tests Comparing Expert Ratings Between PE and non-PE Groups Under Each Protocol and Dimension

| Protocol | Dimension | Group 1 | Group 2 | N1 | N2 | Statistic | Raw p | Adjusted p (Bonferroni) |
|---|---|---|---|---|---|---|---|---|
| Detailed | Effectiveness | non-PE | PE | 24 | 20 | 138 | 0.016 | 0.16 |
| Detailed | Feasibility | non-PE | PE | 24 | 20 | 220 | 0.621 | 1 |
| Detailed | Personality | non-PE | PE | 24 | 20 | 186 | 0.206 | 1 |
| Detailed | Safety | non-PE | PE | 24 | 20 | 168 | 0.0833 | 0.833 |
| Detailed | Total | non-PE | PE | 24 | 20 | 181 | 0.168 | 1 |
| Simple | Effectiveness | non-PE | PE | 24 | 20 | 182 | 0.176 | 1 |
| Simple | Feasibility | non-PE | PE | 24 | 20 | 292 | 0.213 | 1 |
| Simple | Personality | non-PE | PE | 24 | 20 | 200 | 0.355 | 1 |
| Simple | Safety | non-PE | PE | 24 | 20 | 262 | 0.61 | 1 |
| Simple | Total | non-PE | PE | 24 | 20 | 238 | 0.981 | 1 |

Note: Statistic refers to the Wilcoxon test statistic; Raw p represents the unadjusted p-value; Adjusted p (Bonferroni) refers to p-value adjusted for multiple comparisons using the Bonferroni correction method.

**SUPPLEMENT 3.** Results of Friedman Tests for Differences Among Four Training Programs Within Each Protocol

| Protocol | Dimension | Chi_sq(df) | Raw p | Adjusted p (Bonferroni) |
|---|---|---|---|---|
| Detailed | Effectiveness | 4.64(3) | 0.2 | 1 |
| Detailed | Feasibility | 7.72(3) | 0.0521 | 0.521 |
| Detailed | Personality | 9.95(3) | 0.019 | 0.19 |
| Detailed | Safety | 6.20(3) | 0.102 | 1 |
| Detailed | Total | 7.08(3) | 0.0693 | 0.693 |
| Simple | Effectiveness | 6.24(3) | 0.101 | 1 |
| Simple | Feasibility | 1.45(3) | 0.694 | 1 |
| Simple | Personality | 4.10(3) | 0.251 | 1 |
| Simple | Safety | 1.58(3) | 0.665 | 1 |
| Simple | Total | 2.27(3) | 0.518 | 1 |

Note: Chi_sq(df) reflects Friedman chi-square statistic with degrees of freedom; Raw p refers to unadjusted p-value obtained from the Friedman test; Adjusted p (Bonferroni) refers to p-value adjusted for multiple comparisons using the Bonferroni correction method.

**Supplement 4**

**Participant:**
Please help my 15-year-old son design a one-month weight loss and fitness training plan

**ChatGPT 4.0:**
Of course! To effectively help your 15-year-old son design a one-month fat-loss fitness plan, I'll first ask a few questions. These help me provide a more personalized and practical training arrangement:

1. What is his current height and weight?
2. What is his physical condition? (e.g., any chronic issues, joint problems, or exercise restrictions?)
3. What kind of physical activity does he normally do? Is he generally inactive or does he have habits like playing basketball or running?
4. What does his daily routine look like? (e.g., school workload, sleep, how much time he spends sitting, and screen time)
5. What is your main goal? Weight loss, muscle gain, overall fitness improvement, or health?

You can reply with answers to those questions, and I'll tailor a more detailed plan including:
– Weekly training schedule (e.g., aerobic + strength + flexibility)
– Daily training contents (e.g., warm-up, training types, duration)
– Dietary advice (if needed)
– Cautions (to avoid overtraining or injury)

If you're short on time, you can also just tell me the height, weight, and your overall goal. I can first provide a general plan for reference.
**Participant:**
Weekly training schedule and specific content

**ChatGPT 4.0:**
Great! I will design a one-month training plan (4 weeks) for your 15-year-old son that is suitable for teenagers to lose fat and shape their bodies. The goal is to "lose fat + enhance physical fitness + shape your body". It is suitable for boys in junior and senior high school, who are in good health and have no special diseases.
Next is the specific training plan……

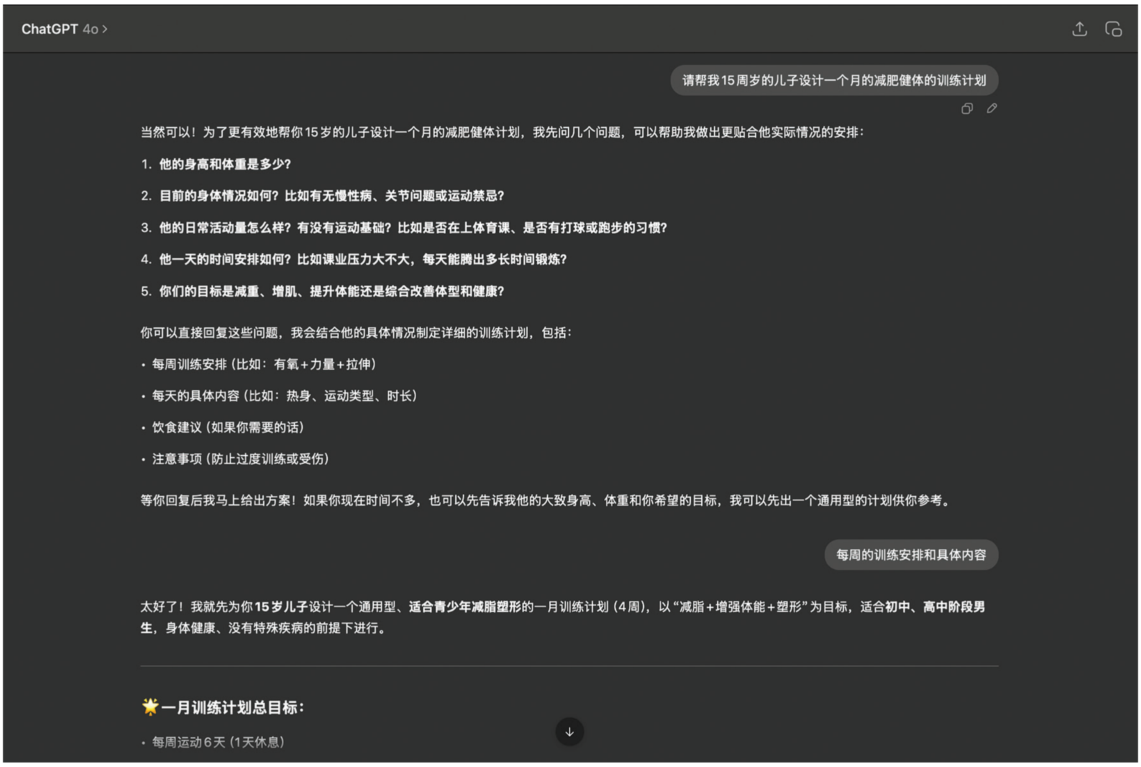

**FIG. 1.** Supplement 1 screenshot

**Supplement 5**

**Participant:**
Why is the generated table different from the one you sent me?

**ChatGPT 4.0:**
Great question! Indeed, the structure of the training schedule I initially provided is based on the FITT principle, whereas the Excel version I generate is a detailed, daily "checklist-style" calendar. So the format is different:

✅ The first format (FITT principle table) is:
– Organized weekly (e.g., Week 1–2, Week 3–4)
– Tailored by training purpose and types
– Emphasizes the 4 FITT components: Frequency, Intensity, Time, and Type
– Serves as a general "training guide framework"

✅ The Excel version is:
– Organized by daily dates
– Better suited for tracking and daily logging
– Emphasizes exact activities, duration, key points
– Easy to print and implement

If you would like an Excel version formatted by the "FITT Four Elements + Training Plan" structure, I can regenerate one like the table below:
Next is the specific training plan…….

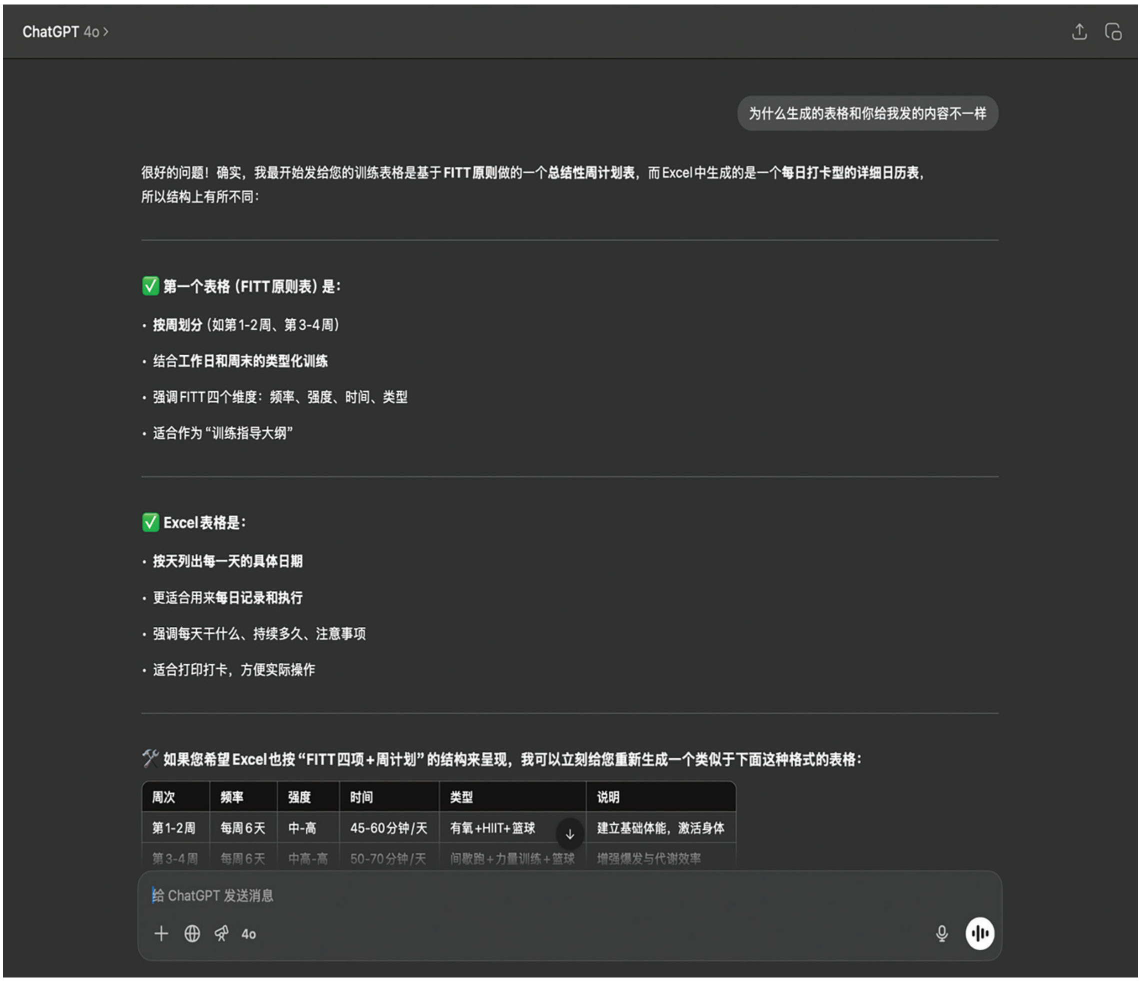

**FIGURE 2.** Supplement 2 screenshot

Articles published in the Biology of Sport are licensed under an open access Creative Commons CC BY 4.0 license.
